# Sexual-biased necroinflammation is revealed as a predictor of bevacizumab benefit in glioblastoma

**DOI:** 10.1093/neuonc/noae033

**Published:** 2024-02-27

**Authors:** Sara Hiller-Vallina, Lucia Mondejar-Ruescas, Marta Caamaño-Moreno, Blanca Cómitre-Mariano, Denisse Alcivar-López, Juan M Sepulveda, Aurelio Hernández-Laín, Ángel Pérez-Núñez, Berta Segura-Collar, Ricardo Gargini

**Affiliations:** Instituto de Investigación Biomédicas I+12, Hospital Universitario 12 de Octubre, Madrid, Spain; Pathology and Neurooncology Unit, Hospital Universitario 12 de Octubre, Madrid, Spain; Instituto de Investigación Biomédicas I+12, Hospital Universitario 12 de Octubre, Madrid, Spain; Pathology and Neurooncology Unit, Hospital Universitario 12 de Octubre, Madrid, Spain; Instituto de Investigación Biomédicas I+12, Hospital Universitario 12 de Octubre, Madrid, Spain; Pathology and Neurooncology Unit, Hospital Universitario 12 de Octubre, Madrid, Spain; Instituto de Investigación Biomédicas I+12, Hospital Universitario 12 de Octubre, Madrid, Spain; Pathology and Neurooncology Unit, Hospital Universitario 12 de Octubre, Madrid, Spain; Instituto de Investigación Biomédicas I+12, Hospital Universitario 12 de Octubre, Madrid, Spain; Pathology and Neurooncology Unit, Hospital Universitario 12 de Octubre, Madrid, Spain; Instituto de Investigación Biomédicas I+12, Hospital Universitario 12 de Octubre, Madrid, Spain; Medical Oncology, Hospital Universitario 12 de Octubre, Madrid, Spain; Instituto de Investigación Biomédicas I+12, Hospital Universitario 12 de Octubre, Madrid, Spain; Pathology and Neurooncology Unit, Hospital Universitario 12 de Octubre, Madrid, Spain; Instituto de Investigación Biomédicas I+12, Hospital Universitario 12 de Octubre, Madrid, Spain; Department of Neurosurgery, 12 de Octubre University Hospital (i+12), Madrid, Spain; Instituto de Investigación Biomédicas I+12, Hospital Universitario 12 de Octubre, Madrid, Spain; Pathology and Neurooncology Unit, Hospital Universitario 12 de Octubre, Madrid, Spain; Instituto de Investigación Biomédicas I+12, Hospital Universitario 12 de Octubre, Madrid, Spain; Pathology and Neurooncology Unit, Hospital Universitario 12 de Octubre, Madrid, Spain; Medical Oncology, Hospital Universitario 12 de Octubre, Madrid, Spain

**Keywords:** bevacizumab, estrogen receptor, glioblastoma, necroinflammation, sex differences

## Abstract

**Background:**

Glioblastoma (GBM) is a highly malignant brain tumor that affects men more often than women. In addition, the former shows a poorer survival prognosis. To date, the reason for this sex-specific aggressiveness remains unclear. Therefore, the aim of this study is to investigate tumor processes that explain these sex differences.

**Methods:**

This was a retrospective study of GBM patients which was stratified according to sex. A cohort with 73 tumors was analyzed with immunohistochemistry, RNA-seq and RT–qPCR to characterize differences in vascular and immunological profiles. Transcriptomic profiling, gene set enrichment analysis, and pathway enrichment analysis were used for discovering molecular pathways predominant in each group. We further investigated the therapeutic effect of bevacizumab (vascular endothelial growth factor A (VEGFA) blocking antibody) in a retrospective GBM cohort (36 tumors) based on sex differences.

**Results:**

We found that under hypoxic tumor conditions, 2 distinct tumor immuno-angiogenic ecosystems develop linked to sex differences and ESR1 expression is generated. One of these subgroups, which includes male patients with low ESR1 expression, is characterized by vascular fragility associated with the appearance of regions of necrosis and high inflammation (called necroinflamed tumors). This male-specific tumor subtype shows high inflammation related to myeloid-derived suppressor cells infiltration. Using this stratification, we identified a possible group of patients who could respond to bevacizumab (BVZ) and revealed a genetic signature that may find clinical applications as a predictor of those who may benefit most from this treatment.

**Conclusions:**

This study provides a stratification based on the sexual differences in GBM, which associates the poor prognosis with the presence of immunosuppressive myeloid cells in the necrotic areas. This new stratification could change the current prognosis of GBM and identifies those who respond to BVZ treatment.

## Graphical Abstract

**Graphical Abstract:**
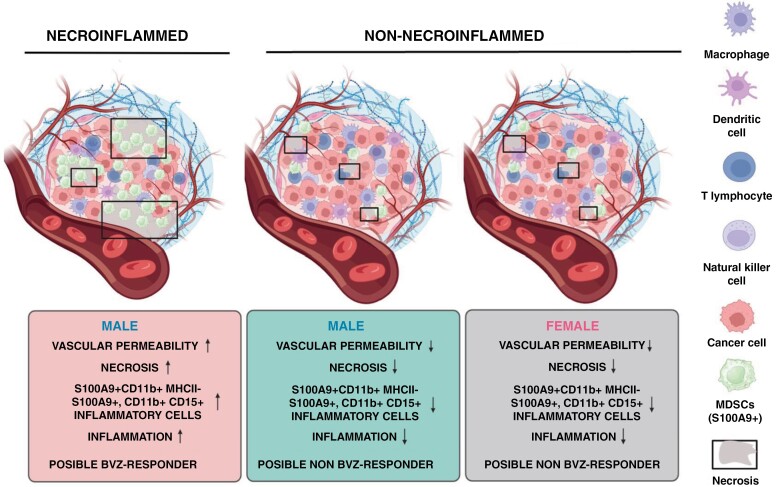

**Key Points**

- Two immuno-angiogenic tumor ecosystems are generated linked to the patients sex and ESR1 expression in GBM.
- The presence of CD11B^+^S100A9^+^MCHII^-^ MDSCs in necrotic areas (tumors necroinflamed) are related to prognosis.
- Sex stratification could identify responders to bevacizumab.

**Importance of the Study**

Despite extensive knowledge about sex differences in the incidence and aggressiveness of human diseases, it is still not sufficient to establish sex-specific treatments. Glioblastoma is the most common malignant brain tumor, providing a good example of this. Here we identified sex-specific tumor subtypes in which necrosis and consequent inflammation linked to MDSCs infiltrate are the critical determinants of poorer survival for male patients. The clinical utility of this study is the identification of a group of patients, based on this stratification, who respond to bevacizumab (BVZ) treatment as well as the establishment of a gene signature that could be used as a predictor of the benefit of BVZ.

Glioblastomas (GBM) are the most common primary brain tumors of the central nervous system (CNS) and one of the most devastating pathologies due to the lack of clinical improvements. In the last World Health Organization (WHO) classification (2021), diffuse gliomas are grouped on the basis of the presence of isocitrate dehydrogenase 1/2 (IDH1/2) mutation, establishing the following subtypes of gliomas: oligodendroglioma, IDH mutant (mut) and 1p/19q-codeleted, grades 2 and 3, Astrocytoma IDH mut, which include grades 2 and 3 (previous name lower-grade glioma, LGG), grade 4 IDH mut gliomas and grade 4 IDH wt gliomas, named GBM.^1^ GBMs are categorized based on the increases in cellular atypia, mitotic activity, and the presence of areas of necrosis and/or microvascular proliferation. Despite standard-of-care with maximal surgical resection followed by radiotherapy plus concomitant and adjuvant chemotherapy with temozolomide (TMZ), the prognosis is dismal, with 15 months overall survival and only 5% of patients alive at 5 years.^2^ In contrast, LGG has a better prognosis, with survival rates ranging from 1 to 15 years. Whereas the incidence of LGG is almost identical in males and females, GBM occurs more commonly in males presenting a male-to-female ratio of 1.61:1.^3^ Sex disparities in the incidence and outcome of human disease, not only in GBM, are widely recognized, but also in most cases, they are not sufficiently understood to allow for sex-specific treatment approaches. However, several reports such as those reported by Dr Rubin’s group reveal a strong need for the establishment of clinical stratification dependent on sex differences because these differences influence therapeutic efficacy.^4^ In this regard, sex differences have been found in cell metabolism and in the proliferation or signaling of integrins, which has a strong impact on the therapeutic effect.^5,6^ In addition, differences in the composition of the GBM microenvironment highlight the need for sex-specific immunotherapies.^7,8^

In the context of differences between sexes, neurosteroids, especially estrogen found in higher concentrations in the female brains, may explain the differences observed. The effects of this hormone are mediated by nuclear estrogen receptors, ERα (encoded by the ESR1 gene), and ERβ (by ESR2) which can translocate to the nucleus and directly modify the expression of their target genes. Given that ER expression tends to decrease as glioma malignancy increases,^9^ it could be speculated that this receptor is involved in glioma pathology. GBM is characterized by aberrant vasculature that is triggered mostly by the vascular endothelial growth factor (VEGF), which promotes angiogenesis, vasculogenesis, and vascular mimicry, all mechanisms that usually overlap.^10^ The anti-angiogenic standard treatment for GBM is bevacizumab (Avastin®, Genentech/Roche) (BVZ), an FDA-approved humanized monoclonal antibody that prevents the interaction of VEGFA with its receptors and inhibits downstream signaling pathways.^11^ BVZ reduces vessel leakiness and pressures within the brain tumor, thereby triggering a reduction in vasogenic edema, thus allowing a decrease in corticosteroid administration.^12^ However, some phase 2 and phase 3 clinical trials performed with BVZ in newly diagnosed GBM patients (AVAglio-B021990 and RTOG-0825) failed to demonstrate a significant overall survival advantage.^13^

We found that under hypoxic tumor conditions, abundant VEGFA is released, generating 2 distinct tumor immuno-angiogenic ecosystems in male patients when stratified based on high or low ESR1 expression. In addition, we identified that one of these subpopulations of males with low ESR1 expression benefits from BVZ treatment. In conclusion, this study provides a coherent view of sex differences in GBM biology and their clinical ramifications and it supports the development of diagnostics and treatments that incorporate sex differences in GBM biology.

## Materials and Methods

### Human Samples

This study included 2 retrospective cohorts of GBM patients (Supplementary Table S2). One of them was composed of 73 patients for the discovery analysis. The other cohort was a highly homogenized cohort of 36 patients who received BVZ after the progression to standard treatment (second line of treatment). All of them were diagnosed at “Hospital 12 de Octubre” (Madrid, Spain) from 2012 to 2022 and have been reclassified according to the current CNS WHO criteria of 2021. Fresh-frozen, formalin-fixed paraffin-embedded (FFPE) tumor samples and clinic-pathological characteristics, including age, sex, tumor histologic type, treatment, recurrence, and death status were collected. Glioma tissues (fresh frozen or embedded in paraffin) were obtained after the patient’s written consent and with the approval of the Ethical Committees of “Hospital 12 de Octubre” (CEI_14/023 and CEI_18/024).

### Patient-Derived Tumor Fragments

Patient-derived tumor fragments (PDTFs) cultures from GBM patients were prepared by cutting 1mm sections of fresh tumor tissue, cultured in collagen with a matrigel mixture, as described in.^14^

### Human Glioma Cells

The Human cells were derived from surgical specimens obtained from patients under treatment at “Hospital 12 de Octubre” in Madrid, Spain.

### Intracranial Tumor Formation and Treatment

The experimental protocols were approved by the Animal Care and Use Committee of the UAM and Comunidad de Madrid (PROEX 183.4/22). Experiments were carried out only with a PDX1 cell line derived from a male patient due to its high necroinflammation values, similar to those patients’ lines comprising the cohorts in this study.

To establish orthotopic xenografts and allografts within the brain, 300 000 cells from PDX1 were resuspended in 2 μL of stem cell culture medium using a Hamilton syringe and injected later in nude male mouse brains. Specifically, the injections were carried out in the striatum, with specific coordinates relative to Bregma (*A*–*P*: -0.5 mm, *M*–*L*: +2 mm, *D*–*V*: -3 mm), utilizing a Stoelting Stereotaxic device. The animals were humanely sacrificed upon the onset of symptoms. For the treatment, animals were treated with BVZ (Zirabev, Pfizer) (10 µg/mL) and isotype control (Bio-Legend, 401401) at a dose of 16 mg/kg 2 times per week through intraperitoneal injection.

### Immunofluorescence (IF) and Immunohistochemical (IHC) Staining

Slides were heated at 60°C for 1 h followed by deparaffinization and hydration, washed with water, and placed into antigen retrieval solution (pressure cooking) in 10 mM sodium citrate pH 6.0. Paraffin sections were permeabilized with 1% Triton X-100 (Sigma) in PBS and blocked for 1 h in PBS with 5% BSA (Sigma), 10% FBS (Sigma) and 0.1% Triton X-100 (Sigma). The primary antibodies (detailed in Supplementary Material) were incubated O/N at 4°C. The second day, sections were washed with PBS 3 times prior to incubation with the appropriate secondary antibody (anti-mouse/rabbit-Dylight 488, anti-mouse/Rabbit/Goat-Cy3, anti-mouse/rat-Cy5, Jackson-Immunosearch) (1:200 dilution) for 2 h at room temperature. Before coverslip application, nuclei were counterstained with DAPI, and imaging was done with a Leica THUNDER microscope. Otherwise, IHC sections were incubated with biotinylated secondary antibodies (1:200 dilution). Target proteins were detected with the ABC Kit and the DAB kit (Vector Laboratories).

### qRT–PCR Assay

RNA was extracted from the tissue using an RNA isolation Kit (Roche). Total RNA (1 µg) was reverse transcribed with PrimeScript RT Reagent kit (Takara). Quantitative real-time PCR was performed using the Light Cycler 1.5 (Roche) with the SYBR Premix Ex Taq (Takara). The primers used for each reaction are indicated in Supplementary Table 3. Gene expression was quantified by the delta–delta *Ct* method (more detailed in Supplementary Material).

### Bioinformatics Studies

The Cancer Genome Atlas (TCGA) Pan-Cancer (PANCAN), GBM, LGG, and GBM + LGG dataset was accessed via cBioPortal (https://www.cbioportal.org/), UCSC xena-browser (https://xenabrowser.net), and Gliovis (http://gliovis.bioinfo.cnio.es) for extraction of the data: overall survival, gene’s expression level, and the distribution of the different genetic alterations. GBM cohort from Rembrandt, Leey, and CGGA dataset was accessed via Gliovis.

### RNA-seq Assay

#### Gene expression quantification.

—STAR was used to obtain the number of reads associated with each gene in the Gencode v31 annotation (restricted to protein-coding genes, antisense, and lincRNAs). Raw counts for each sample were imported into R statistical software. The extracted count matrix was normalized for library size to compute CPM expression levels.

### Statistical Analysis

For the statistical analysis of the samples, no specific statistical method was used to predetermine the sample size, but the sizes of our cohort are similar to or greater than those reported in previous publications, using GraphPad Prism 8.0 software.^15–17^ The normality of the data distribution (Kolmogorov–Smirnov test) and the homoscedasticity were formally tested. Nonparametric unpaired or paired tests (Mann–Whitney or Wilcoxon test) or parametric with equal or unequal variance (Student’s *t* and Welch’s correction test) were used. *P < .05, **P < .01, ***P < .001, ****P < .0001 were considered significant. The area under the receiver operating characteristic (ROC) curve (AUC) was calculated for necroinflammation gene signature to determine the diagnostic performance for the 2 male GBM subtypes and GBM females. For Kaplan–Meier survival curves, the level of significance was determined by the 2-tailed log-rank test. For correlation analysis between each gene, expression data were tested by Pearson’s correlation coefficient. Precise experimental details are provided in the figure legends.

Additional information can be found in Supplementary Materials and Methods.

## Results

### Sex Disparities in Pan-Cancer and Glioblastoma

Sex differences are evident in tumor incidence in many different cancer types, affecting males more than females, such as hematological malignancies, as well as cancers of the bladder, colon, skin, liver, and brain.^18^ However, cancer death rates based on sex disparities vary by cancer type. Based on *in silico* study of the Pan-Cancer dataset from the TCGA showed that, as expected, males develop cancer more often but surprisingly it was only significant for glioblastoma and Head and Neck carcinoma (Supplementary Table 1). When we focused on analyzing the sex differences implication in glioma survival (in astrocytoma excluding oligodendroglioma tumors), we observed a significant correlation of male patients with lower survival in the glioma cohort (TCGA diffuse glioma) (Supplementary Figure 1A), but when we considered Astrocytoma IDHmut (grades 2–4) (Supplementary Figure 1B), and GBM (grade 4, IDHwt) separately (Supplementary Figure 1C), we only observed the male implication in the last cases (GBM IDHwt). It should be noted that the analysis of the average initial Karnofsky Performance Status (KPS) between male and female patients with GBM did not show significant differences (Supplementary Figure 1D). This result suggests that men are more sensitive to some tumor processes involved in glioma aggressiveness, in agreement with the data of Barnholtz–Sloan´s group.^19^ We then analyzed the mutational frequency in groups based on sex, but we found no significant enrichment in any genes that could explain the difference in patient survival, neither glioma diffuse nor GBM cohort (Supplementary Figure 1E and F).

### Tumoral ESR1 Expression was Associated with Overall Survival and Stratified Patients with GBM

Many neurological diseases like stroke, brain injury, and traumatic brain also show sex differences, which are most likely attributed to the expression of estrogen and estrogen receptors (ER) in reactive astrocytes.

**Figure 1.**
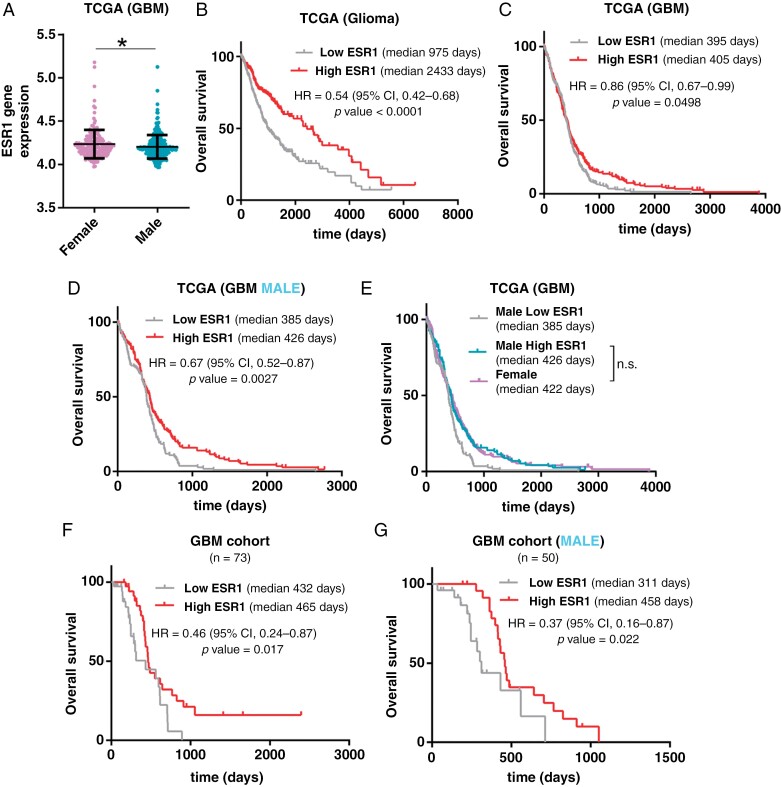
ESR1 expression correlates inversely with tumor aggressiveness in male GBM patients. (**A**) ESR1 gene expression analysis by RNA-Seq in GBM cohort (TCGA GBM IDH wt) grouped according to sex. (**B and D**) Kaplan–Meier overall survival curves of patients from the glioma cohort (TCGA GBM + LGG) (*n* = 697) (**B**), GBM cohort (TCGA GBM IDH wt) (*n* = 474) (**C**), and GBM male cohort (*n* = 288) (**D**). Patients in each cohort were stratified into 2 groups based on high and low ESR1 expression values. (**E**) Kaplan–Meier overall survival curve from the GBM cohort (TCGA GBM IDH wt) (*n* = 474) stratified in 3 subgroups male high ESR1, male low ESR1, and female. (**F and G**) Kaplan–Meier overall survival curves of patients from our own GBM cohort (*n* = 73) (**F**) and only male patients in our own GBM cohort (*n* = 50) (**G**). Patients in each cohort were divided into 2 groups based on high and low ESR1 gene expression values, measured by qRT–PCR analysis. The data are represented with means ± SEM. The statistical significance was assessed using the Student’s *t*-test. Kaplan–Meier curves were compared using the log-rank test (Mantel–Cox). *P ≤ .05; **P ≤ .01; ***P ≤ .001, ****P ≤ .0001. n.s., not significant.

To determine if tumor ER expression, both α (ESR1) and β (ESR2), plays a role in brain tumor protective function, we performed an analysis of gene expression in the GBM dataset. We observed that the transcription of ESR1 is increased in female compared to male patients (Figure 1A), without differences in ESR2 and androgen receptor (AR) expression based on sex (Supplementary Figure 2A and B). Moreover, the expression of ESR1 correlated with overall survival in glioma (TCGA glioma diffuse cohort) (Figure 1B) and GBM TCGA cohort (Figure 1C), but with an inverse correlation in Astrocytoma IDHmut (Supplementary Figure 2C), showing the same affectation as when we sub-grouped the patients by sex. As expected, we found a positive association between ESR1 expression and overall survival in other GBM cohorts from Rembrandt and Leey dataset (Supplementary Figure 2D and E). On the other hand, we did not observe any implication in overall survival based on ESR2 or AR expression (Supplementary Figure 2F and G). To our surprise, if we stratified only female patients with GBM into 2 groups based on high and low ESR1 expression values we did not observe any implication in survival (Supplementary Figure 2H), but if we stratified the male patients we observed that a higher transcription of this gene was associated with an increased in overall survival (Figure 1D and Supplementary Figure 2I), becoming survival rate in men with high transcription of ESR1 similar to female patients (Figure 1E and Supplementary Figure J).

To validate these results, we first confirmed in our own glioma cohort the survival benefit in female versus male patients (Supplementary Figure 2K–L). Then, we classified our own GBM cohort into 2 groups based on ESR1 expression, measured by qRT–PCR. We observed that high ESR1 expression is associated with less aggressive behavior of GBM (Figure 1F), even when we only considered male GBM patients (Figure 1G). A similar glioma patient’s prognosis classification was obtained when we measured ESR1 by immunohistochemical (IHC) staining on glioma samples (Supplementary Figure 2M). Based on this result, our study addressed the impact of ESR1 expression in the male GBM cohort to understand the reason for higher tumor aggressiveness in a subgroup of male patients compared to the rest of the males and all females with glioma.

### Stratification Based on ESR1 Expression Was Associated With Normalization of the GBM Tumor Vessel

**Figure 2.**
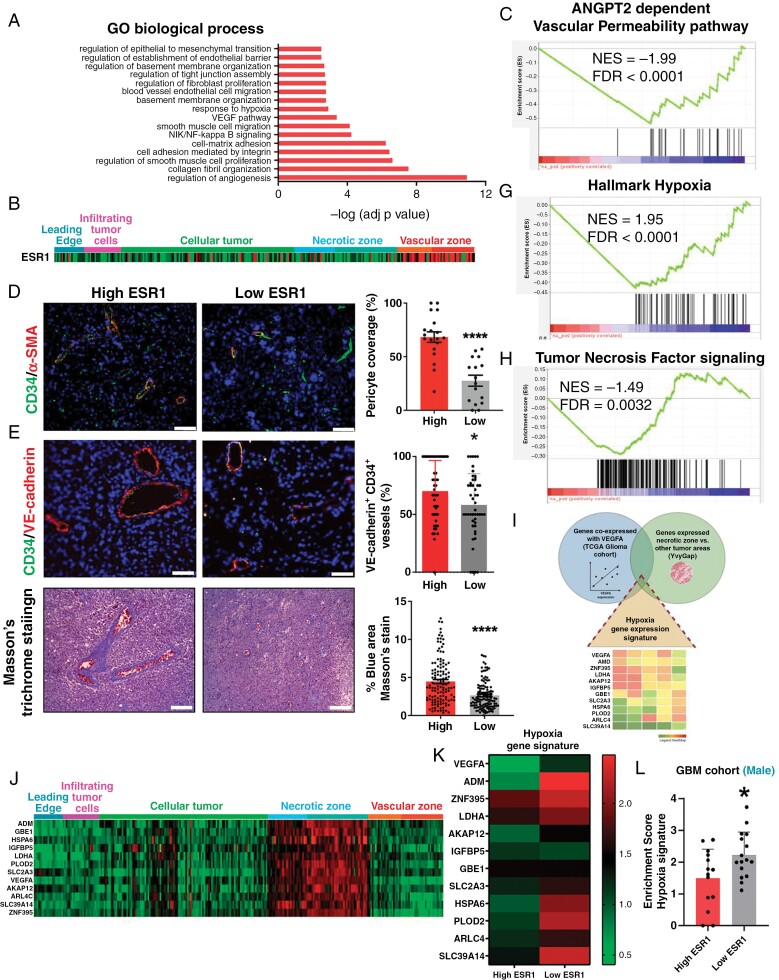
Increased ESR1 levels were associated with vasculature normalization in GBM tumors and decreased hypoxia. (**A**) Gene ontology analysis (DAVID Gene Ontology program) of biological processes based on 1000 genes coexpressed with ESR1 in the TCGA GBM cohort. (**B**) Heatmap of ESR1 expression in different areas: leading edge, infiltrating tumor cell, cellular tumor, necrotic, and vascular areas, using the IvyGAP dataset (Ivy GBM Atlas Project). (**C**) GSEA enrichment plot analysis using the expression values of ESR1 coexpressed genes as a template and ANGPT2-dependent vascular permeability pathway gene set from the Biocarta pathway database. (**D**–**F**) Representative IF images and quantification of CD34/α-SMA (**D**) and CD34/VE-Cadherin (**E**) costaining, and Masson’s trichrome IHC staining (**F**) performed in our own GBM cohort grouped according to their ESR1 levels (*n* = 10). (**G and H**) GSEA enrichment plot analysis using the expression values of ESR1 coexpressed genes as a template and Hypoxia hallmark (**G**) and tumor necrosis factor signaling (**H**) gene set from the Biocarta pathway database. (**I**) Establishment of hypoxia gene expression signature and representative Venn Diagram showing genes coexpressed with VEGFA and genes expressed in necrotic zones. (**J**) Heatmap showing common genes coexpressing with VEGFA (TCGA GBM IDH wt cohort) that are present exclusively in necrotic areas of the tumor using the IvyGap dataset. (**K and L**) Heatmap (**K**) and quantification (**L**) of hypoxia gene signature expression in our male GBM cohort (*n* = 30) stratified into high and low ESR1 levels. The data are shown as means ± SD. The IF and IHC statistical analyses were performed using the Student’s t-test and the Mann–Whitney test. The significance of gene expression was analyzed using a Student’s *t*-test. *P ≤ .05; **P ≤ .01; ***P ≤ .001, ****P ≤ .0001. n.s., not significant. Scale bar 50 μm.

To obtain information on the reduction of tumor aggressiveness in stratification by ESR1, we performed a DAVID_GO analysis (a functional annotation clustering tool) to search for the pathways co-upregulated with this gene in GBM. We found a positive association between *ESR1* expression and angiogenesis regulation processes. Some of the ESR1-associated pathways were collagen fibril organization, pericyte migration, cell adhesion (by integrin and tight junction), response to hypoxia, and VEGF signaling (Figure 2A). According to ESR1 expression, we observed ESR1 was upregulated in vascular tumor zones using the IvyGAP (Ivy GBM Atlas Project) dataset analysis (Figure 2B). In addition, we observed that ESR1 transcription negatively correlated with the expression of angiopoietin 2 (ANGPT2)-dependent vascular permeability pathway (Figure 2C). The ANGPT2-Tie2 axis has emerged as a regulator of tumor angiogenesis,^20^ due to it exhibiting high expression in human tumor vascular remodeling as well as very low expression in normal tissue. ANGPT2 acts as a destabilizer for tumor vasculature, generating vascular leakage and pericyte dropout from the glioblastoma vasculature.^17,21^

In our analyses of structural and functional changes of blood vessels in the tumor, we found that male GBM patients with high-ESR1 expression are associated with an increase in αSMA pericyte coverage on tumor vessels (40.68-fold, Figure 2D), endothelial tight junctional molecule VE-cadherin (12.02-fold, Figure 2E) and distribution of collagen type IV + basement membrane coverage (1.68-fold, Figure 2F) on tumor vessels. Collectively, these results indicate that ESR1 expression stratifies tumors based on their vasculature, presenting high-ESR1 tumors with a normalized vasculature which is observed in GBM with the best prognosis, as we have recently shown.^15,17^

### GBM Tumors With Low ESR1 Expression Were Characterized by Increased Hypoxia, Necrotic Areas and Extensively Infiltrated Myeloid Cells

**Figure 3.**
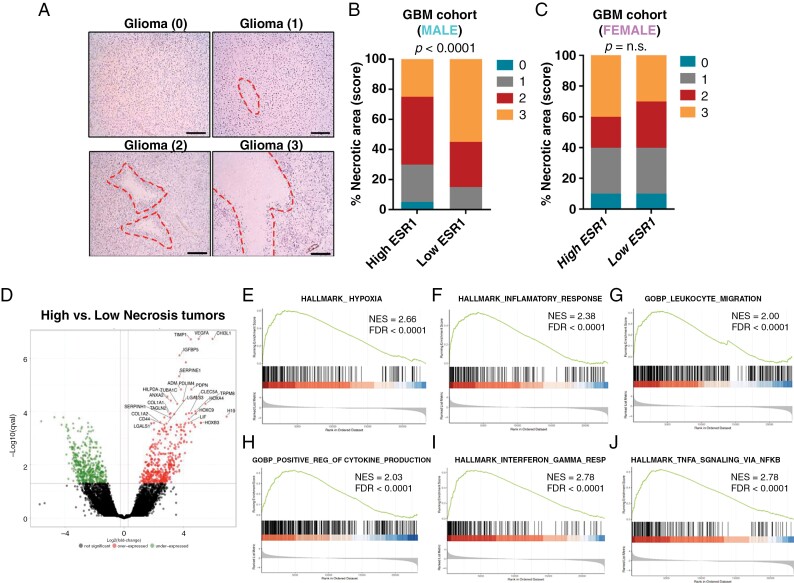
Low ESR1 expression GBM tumors were characterized by augmented necrotic areas and an increased expression in inflammation pathways signatures. (**A**) Representative IHC images stained with hematoxylin and eosin representing the corresponding SCORE (0–3) used for necrosis levels graduation in our own GBM cohort patients. Necrotic areas are highlighted with a red line and the necrotic area score is represented between brackets. (**B and C**) Quantification of necrosis levels in male (*n* = 35) (**B**) and female (*n* = 16) (**C**) GBM cohort patients grouped according to the ESR1 levels. (**D**) Volcano plot showing differential gene expression in GBM comparing high necrotic tumors with low necrotic tumors in our own GBM cohort (data obtained by RNA-seq analysis, *n* = 4 per group). (**E**–**J**) GSEA enrichment plot analysis using expression values of genes upregulated in necrotic tumors as a template and Hypoxia hallmark (**E**), Tumor inflammatory response hallmark (**F**), Leukocyte migration gene ontology biological process (**G**), Positive regulation of cytokine production gene ontology biological process (**H**), Interferon-gamma response hallmark (**I**) and TNFα signaling via NFKB hallmark (**J**) gene set from the Biocarta pathway database.

The disruption of the vasculature has been associated with the induction of hypoxic pathways in tumors.^15,22^ Accordingly, we observed that ESR1 exhibited a strong negative correlation with a hallmark hypoxia signature and with tumor necrosis factor signaling (Figure 2G and H). Tumors with accelerated tumor growth that exceed vascular supply, like GBMs and other solid tumors, are characterized by extensive hypoxia tissue which unleashes the development of necrotic processes.^23^ Necrosis in GBM is of particular interest since it is a diagnostic hallmark and positively correlates with tumor aggressiveness and poor outcomes.^24,25^ To validate the results in our own male GBM cohort, we first defined a hypoxia signature using the IvyGAP to select the most relevant genes included in the VEGFA pathway signature that were upregulated in the perinecrotic and pseudopalising-cell-necrosis tumor zones (Figure 2I and J). Notably, this signature was increased in low ESR1 tumors compared to high ESR1 expression in male GBM patients (Figure 2K and L). GBM with a compromised vasculature with vessel leakage and hypoxia present extent of necrosis areas.^15^ This observation was confirmed by histologic analysis of tumor sections (Figure 3A). It showed more extensive necrotic areas in low ESR1 compared with high ESR1 tumors in the male GBM cohort (Figure 3B) and no differences in female patients according to ESR1 expression. (Figure 3C)

Transcriptional analysis by RNA-seq of tumors with low and high necrosis scores reflects drastic changes (Figure 3D), which lead to an increase in inflammation and immune recruitment, linked to hypoxia (Figure 3E–J). This transcriptional profile favors the recruitment of immune cells that can promote an excess of inflammatory processes with toxic brain consequences such as the secretion of INFγ or TNFα, among other inflammatory interleukins (Figure 3F–J). These results suggest that low ESR1 tumors present vascular fragility which seems to be associated with the appearance of areas of necrosis and hypoxia. The mechanisms by which necrosis implicates glioma progression remain unclear, but it is known that necrosis plays a role in reshaping the local brain tumor microenvironment (TME) by releasing endogenous damage-associated molecular patterns (DAMPs), capable of recruiting tumor-associated macrophages (TAMs), which could facilitate disease progression^26^

### Necrosis Drives a Chronic Inflammatory State in the GBM Microenvironment

To understand the mechanism of the necrosis role of aggressiveness, we performed a differential gene expression analysis between the necrotic zone and the rest of all tumor areas (leading edge, infiltrating tumor cells, cellular tumor, and vascular zone) from the IvyGap dataset. This analysis shows a series of differentially expressed genes that are associated with biological processes such as inflammation, hypoxia, chemotaxis of immune cells, and angiogenesis, among others (Supplementary Figure 3A and B). Within the genes expressed in necrosis areas, we highlight S100A9 (Supplementary Figure 3A), a member of the S100 family which is involved in glioma progression, metastasis, invasion, and so on.^27^ S100A9 is also called a myeloid-related protein and as the name suggests these proteins are specifically highly expressed in myeloid cells, as we can observe in a single-cell RNA-seq analysis of human glioma samples^28^ (Supplementary Figure 3C and D).

**Figure 4.**
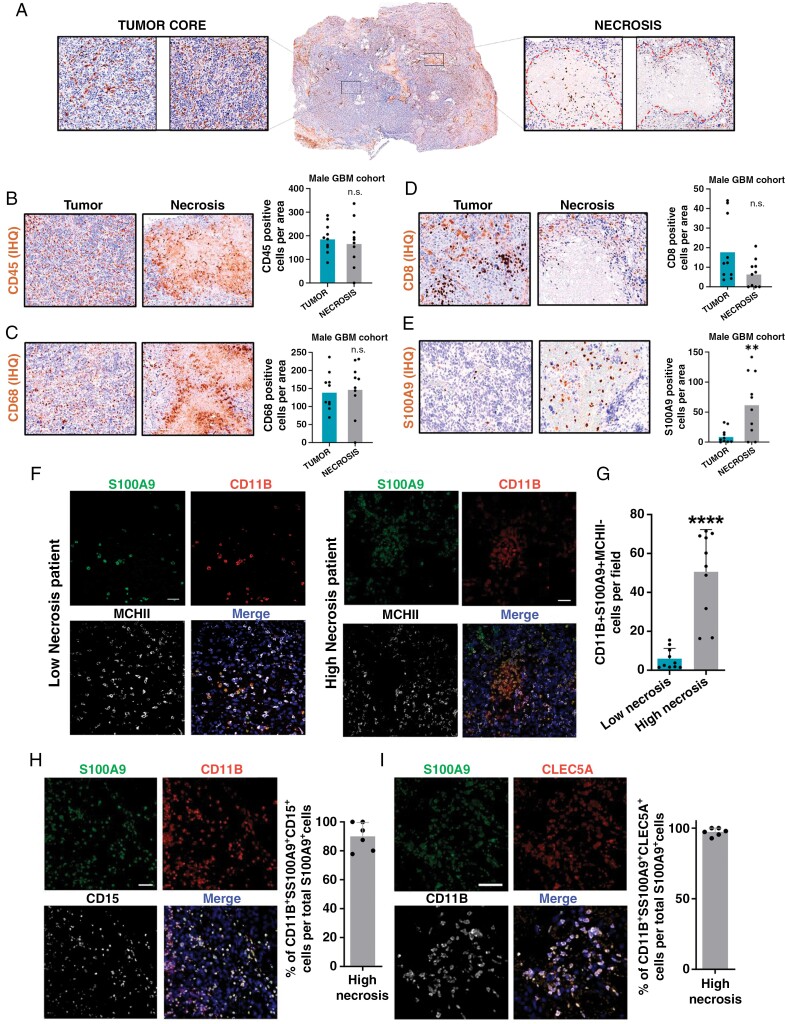
Necrotic areas in GBM recruit myeloid suppressor cells positive for S100A9. (**A**) Representative IHC of tumor core and necrosis areas from patient’s tissue. (**B**–**E**) Representative IHC image and quantification of CD45 (**B**), CD68 (**C**), CD8 (**D**), and S100A9 (**E**) positive cells per field in each region, tumor core and Necrosis areas in our male GBM cohort(*n* = 10 per group). (**F**–**G**) Representative IF (**F**) and quantification (**G**) of CD11b^+^S100A9^+^MCHII^-^cells from male GBM cohort according to necrosis levels (*n* = 10 per group). (**H** and **I**) Representative IF and quantification of the percentage of CD11b^+^S100A9^+^CD15^+^triple-positive cells (H) and CD11b^+^S100A9^+^CLEC5A^+^ per total S100A9^+^ cells from the male GBM cohort categorized as tumors with high necrosis (*n* = 6). The data are represented with mean ± SEM. The statistical IHC and IF significance was determined using the Mann–Witney test. *P ≤ .05; **P ≤ .01; ***P ≤ .001, ****P ≤ .0001. n.s., not significant. Scale bar 300μm (IHC) and 50 μm (IF).

To confirm their identity, firstly we performed IHC with standard immune cell markers (CD45, CD68, and CD8) and S100A9 on tumor sections, distinguishing tumor core and necrotic areas (Figure 4A–E). Surprisingly, only S100A9 positive cells are specifically and significantly observed in necrotic areas (Figure 4E), and thus we observed a significantly large infiltration of CD11B^+^S100A9^+^MCHII^-^ immunosuppressive myeloid cells (MDSCs) in tumors with high necrosis compared to low necrosis male GBM tumors (Figure 4F and G). In line with the recent phenotypic classification of MDSCs, we also show a label of this type of cell with CD11B^+^S100A9^+^CD15^+^(Figure 4H). In addition, we identified by single-cell RNA-seq analysis (Supplementary Figure 3E) that S100A9 is coexpressed in myeloid cells with genes that define an immune suppressive profile (a hallmark of GBM microenvironment) such as CLEC5A,^29^ MS4A4A^30^ and TREM1,^31^ one of them confirmed by IF on male GBM tissue (Figure 4I).

On the other hand, damage-associated molecular patterns (DAMPs) release normally initiates inflammation and when unchecked (necrotic damage) can facilitate a chronic inflammatory state^32^ that results in the upregulation of cell survival pathways to compensate for an increasingly dramatic environment. This microenvironmental restructuring that follows necrotic damage induces the differentiation of myeloid cell subpopulations that thrive under these selective pressures. Interestingly, S100 proteins are potent DAMPs that are enriched in brain and glioma tissues.^33^

**Figure 5.**
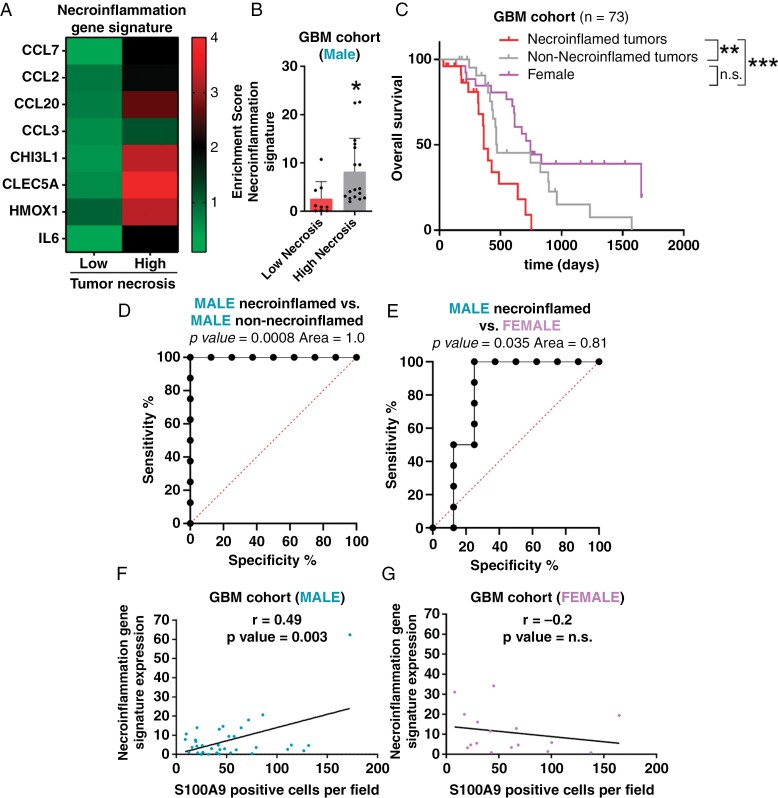
Necroinflammation associated with S100A9 cells defines the aggressiveness of male GBM patients (**A and B**) Heatmap (**A**) and quantification (**B**) of necroinflammation gene signature expression in our male GBM cohort (*n* = 50) stratified into high and low necrosis levels. (**C**) Kaplan–Meier overall survival curves comparing female and male patients stratified according to necroinflammated and non-necroinflammated tumors, from our own GBM cohort (*n* = 73). (**D and E**) ROC curve plotted for diagnostic potential and discriminatory accuracy of necroinflammation gene signature to distinguish male patients (**D**) or necroinflamed male from female patients (E). The corresponding AUC (Area) and *P* value is reported. (**F and G**) Correlation between necroinflammation signature expression and S100A9 positive cells in males (*n* = 35) (**F**) and females (*n* = 16) (**G**) from our own GBM measured qRT–PCR. The data are represented with mean ± SEM. A comparison of Kaplan–Meier curves was performed with a log-rank test (Mantel–Cox). Correlation was performed with the Pearson correlation coefficient. *P ≤ .05; **P ≤ .01; ***P ≤ .001, ****P ≤ .0001. n.s., not significant.

To identify a genetic signature of a link between necrosis and inflammation called “necroinflammation,” we selected the genes upregulated with S100A9 in GBM that were associated with inflammatory processes and at the same time were upregulated in the necrotic zone compared to the rest of the areas in the IvyGap dataset (Supplementary Figure 3F and G). Accordingly, this signature was increased in high necrotic tumors compared with low necrotic tumors (Figure 5A and B).

Together, our results suggest that under hypoxic tumor conditions, abundant proangiogenic factors are released, highlighting VEGFA, which generates 2 different angiogenic phenotypes in male and female GBM patients, linked to sex hormone signaling, especially the expression of ESR1. One of them, which includes mostly men patients (with low ESR1 expression), is characterized by vascular fragility associated with the appearance of areas of necrosis and high inflammation (here called necroinflamed tumors). Whereas the other, including both women and men patients with high ESR1 expression, is characterized by vascular normalization without the other tumor processes (therefore called non-necroinflamed tumors). Importantly, if we stratified the GBM cohort into 3 groups based on sex and necroinflamed phenotype, we observed that necroinflamed male gliomas are the most aggressive tumors compared to female and non-necroinflamed male tumors, both with equally higher survival rates (Figure 5C). This small signature showed 100% prediction in specificity and sensitivity in the male cohort (Figure 5D) and 81% prediction for the differential diagnosis between female and male patients with necroinflamed tumors (Figure 5E), using ROC curves. Thus, these data demonstrate that GBM progression can be characterized by the necroinflammation gene signature. On the other hand, we have been able to establish that the necroinflammation signature positively correlates with the increase in S100A9 cells in male patients (Figure 5F) and not in female patients (Figure 5G).

Taken together, our results show a potential explanation for the poor prognosis observed in a group of men with low ESR1 levels, high necrosis, and an acute inflammation profile, which could have important implications for several aspects of glioma research and clinical practice.

### Necroinflammation-Dependent Response to Bevacizumab

Based on these findings presented here, we hypothesize that the role of VEGFA in GBM is related to inflammation of necrosis rather than to a process of angiogenesis and vascular function. Thus, we investigated the effect of BVZ, a monoclonal antibody that blocks VEGFA, on GBM based on necroinflammation. Bevacizumab was the first antiangiogenic drug to be approved for clinical use and is currently indicated for several cancer types.

**Figure 6.**
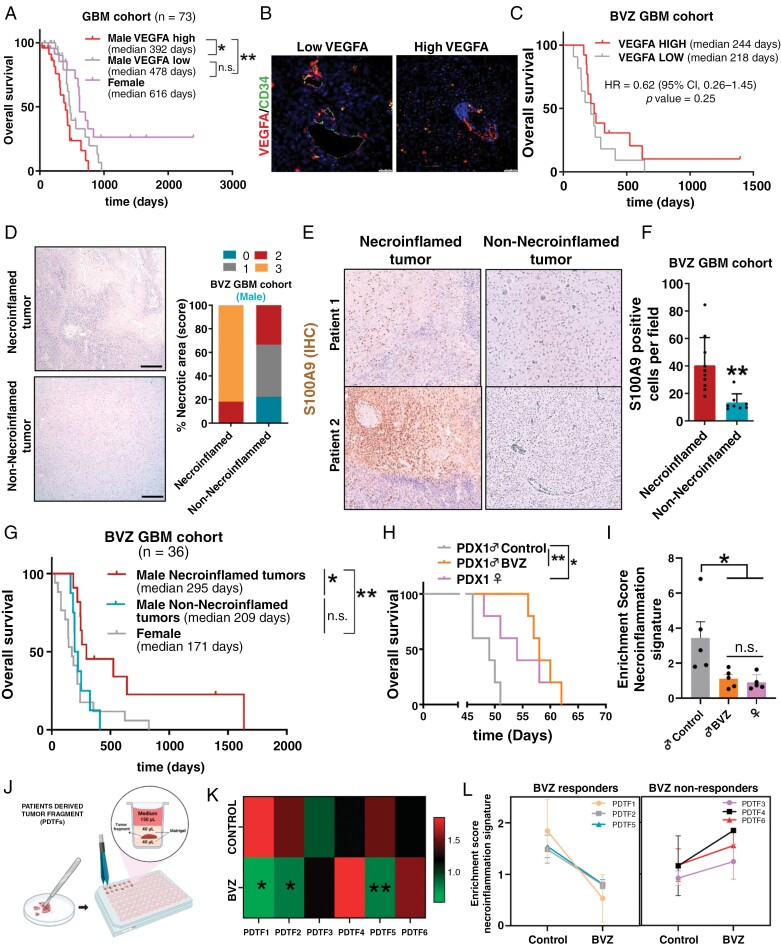
Necroinflammation-dependent response to BVZ treatment. Kaplan–Meier overall survival curves of patients from our own GBM cohort (*n* = 73), stratified into 3 groups based on sex and VEGFA gene expression measured by qRT–PCR. (**B**) Representative images of VEGFA and CD34 IF staining in sections from GBM samples treated with BVZ (*n* = 36). (**C**) Kaplan–Meier overall survival curves of patients from the BVZ GBM cohort (*n* = 36). Patients were stratified into 2 groups based on high and low VEGFA expression. (**D**) Representative pictures of the hematoxylin and eosin staining of different samples from the BVZ GBM cohort. The percentage of tumors with different necrotic area scores is shown on the right. (**E and F**) Representative images (**E**) and quantification (**F**) of S100A9 IHC staining in samples from the BVZ GBM cohort stratified into necroinflamed and non-necroinflamed tumors based on necrosis levels established in Figure 6D (**F**). (**G**) Kaplan–Meier overall survival curves of patients from the BVZ GBM cohort (*n* = 36) stratified into 3 groups male necroinflamed tumors, male non-necroinflamed tumors, and female patients, grouping according to D–F. (**H**) Kaplan–Meier overall survival of mice (female and male) that were orthotopically injected with PDX1 cells and subsequently treated with intraperitoneal injection with BVZ (16 mg/kg 2 days per week, *n* = 6 male mice) (**I**) Quantification of necroinflammation gene signature expression by qRT–PCR in tumors from H (*n* = 5). (**J**) Graphic scheme of the PDTFs procedure. (**K**) Heatmap of necroinflammation gene expression signature in the different PDTF samples. (**L**) Quantification of enrichment score necroinflammation gene expression signature in PDTFs after treatment with BVZ (10 µg/mL during 24 h). The data are represented with mean ± SEM. A comparison of Kaplan–Meier curves was performed by applying the log-rank test (Mantel–Cox). The statistical significance was assessed using Student’s *t*-test. *P ≤ .05; **P ≤ .01; ***P ≤ .001, ****P ≤ .0001. n.s., not significant. Scale bar 50 μm.

To begin this analysis, we first analyzed the implication of VEGFA expression in GBM pathology and survival. Thus, we observed an inverse correlation between the expression of ESR1 and VEGFA in the TCGA and our GBM cohort (Supplementary Figure 4A and B, respectively). The same effect of necroinflammation phenotype on GBM survival was observed with VEGFA expression, where male GBM with high VEGFA expression (equivalent to necroinflamed tumors, Supplementary Figure 4C) is the most aggressive tumors compared to female and male tumors with low VEGFA expression, both with equally higher survival rate (Figure 6A and Supplementary Figure 4C). Collectively, this reinforces the idea of GBM stratification into 3 subtypes based on sex and necroinflamed phenotype. These results could also suggest the possible differential effect in male and female GBM patients after inhibiting VEGFA.

We then study the BVZ efficacy depending on necrosis and inflammation tumor process. A total of 36 patients with GBM at first recurrence following standard therapy (radiation and temozolomide) were recruited to second-line BVZ treatment in this retrospective study. Firstly, we corroborated those high levels of VEGFA expression (measured by IF) (Figure 6B), and this was not associated with better patient outcomes after BVZ treatment when using survival time from the treatment start date (Figure 6C). Based on our stratification surprisingly the male patients who received BVZ therapy with a phenotype of high necrosis (Figure 6D and E) and inflammation linked to high infiltration of S100A9 positive myeloid cells (Figure 6F), obtained a survival benefit (Figure 6G), that is not shown by male patients without this tumor necroinflammation phenotype, nor by female patients. To validate this hypothesis, we treated with BVZ 1 patient-derived xenograft (PDXs)-injected into the brains of immunodeficient male mice. First, we analyzed the expression of necroinflammation gene signature (Supplementary Figure 4E) in a panel of PDX grown in the brain to select the one with the greatest necroinflammation phenotype. We observed that PDX1 cells treated with BVZ grew much slower than the PDX1 cells control (Figure 6H). The effect on decreasing expression of the necroinflammation gene signature was validated by qRT–PCR using human-specific primers (Figure 6I). Together, our results confirmed the specific effect of BVZ on inflammatory processes. In addition, we observed that PDX1 aggressiveness is different depending on the sex of the host mice, with male PDX1 being more aggressive than female PDX1 tumors. Furthermore, it should be noted that female PDX1 tumors have similar survival rates to male PDX1 tumors treated with BVZ (Figure 6H). In addition, the PDX1 implanted in female mice brains presented a decrease in necroinflammation gene expression signature compared to itself grown in male mice (Figure 6H). This evidence suggests that the necroinflammatory phenotype in male hosts are essential in BVZ response.

Finally, to translate the results into clinical settings, we developed an organotypic culture platform using Patient-Derived Tumor Fragments (PDTFs, Figure 6J), as described in.^14^ In this platform, fresh tumor tissue from patients was treated with BVZ for 24 h to evaluate the anti-inflammatory response using the necroinflammation signature gene expression described above. This analysis revealed that BVZ generated a decrease in necroinflammation gene expression signature (measured by RT–qPCR) compared to the control condition only in GBM with necroinflammation phenotype (Figure 6K and L). We then observed that patients who responded to BVZ in the PDFTs assay showed high infiltration of S100A9 positive cells compared to nonresponders to BVZ (Supplementary Figure 4F).

It is important to note that these data provide evidence of the existence of a subgroup of male glioma patients responding to BVZ treatment linked to the anti-inflammatory effect and the establishment of a predictive signature to a positive response to BVZ.

## Discussion

Despite knowledge of the influential role of age and sex in the aggressiveness of glioma, the risk factors associated with the development of this disease are not defined.^3^ There is a greater incidence in men versus women (1.6:1), suggesting a hormonal influence during the development and progression of the disease.^34^ Although the role played by estrogen and its receptors (ESR1 and ESR2), during the development of GBM is still not clear, here we have been able to identify an association of ESR1 with the tumor microenvironment. In accordance, recently other genes such as KDM5D and KDM6A have been associated with the tumor microenvironment based on sex differences for colon cancer and GBM, respectively.^8,35^ These articles emphasize the need to determine sex differences as this greatly affects the response to immunotherapies.

We have found 2 vascular phenotypes based on the high/low ESR1 tumor expression. Low ESR1 tumors are characterized by a hypoxic condition with the instability of the vascular that leads to necrotic areas in the tumors, whereas the second phenotype is induced by high ESR1 expression with vascular integrity and normoxia condition. This alteration could explain the difference in tumor aggressiveness since it is known that the degree of tumor necrosis negatively correlates with GBM patient survival, although it is still unclear how necrosis can decrease overall survival.^24,26^ Necrosis and its cell death entities are related to the initiation of the inflammation process. Certain molecules, which have been dubbed DAMPs or alarmins, are thought to promote inflammation upon release from necrotic cells.^36^ Of interest, DAMPs that are enriched in glioma tissue are adenosine/adenosine triphosphate (ATP), hyaluronan (HA), high-mobility group box 1 (HMGB1), interleukin (IL)-1α, and S100 proteins.^37^ Our *in silico* analysis with the YvyGap dataset showed the upregulation of S100A in the necrotic zone compared to the rest of the tumor areas, confirming the expression linked to the necrotic processes of these genes in glioma. Some examples of DAMP mechanisms of action are how ATP binds to its own receptors on many immune cells, including macrophages, inducing an M2-like immunosuppressive phenotype,^38^ or how HMGB1 acts through TLR4 and the receptor for advanced glycation end products (RAGE) to initiate pro-inflammatory cytokine release, recruiting bone marrow-derived monocytes to tumor site.^39^ In this sense, our analysis revealed that perinecrotic and necrotic zones are infiltrated by S100A9 + myeloid cells, specifically myeloid-derived suppressor cells (MDSC). Thus, several studies demonstrated that S100A9 expression is one of the hallmarks that distinguish MDSCs from the rest of the myeloid population.^40^ In addition, S100A9 was reported highly expressed in many cancers, glioma included,^41^ its correlation with survival of patients^42^ and resistance to immunotherapy.^43^ Thus, a study demonstrated that patients with proliferating monocytic MDSC (mMDSC) were predominant in male tumors and granulocytic MDSC (gMDSC) with IL1β expression correlated with a poor prognosis in female patients.^7^ More recently, increased T-cell exhaustion was observed in male GBM patients, which could better define the immunotherapy applications based on sex differences.^8^

S100A9 is implicated in the activation of NLRP3 inflammasome in response to various pathological conditions such as DAMPs-mediated inflammation.^44^ This activation increased the secretion of pro-inflammatory cytokines (IL-6, IL-1β, and TNFα) and chemokines (IL-8, Gro-α, and MIP-1α/β) that are prevalent in gliomas.^45^ In this report, we have defined a gene expression signature of inflammation linked to S100A9/CLEC5A positive MDSCs whose genes are expressed in necrotic areas of the tumor. This signature, named necroinflammation signature, could have diagnostic, as well as great predictive value, as the different subtypes could have a different sensitivity to antiangiogenic or immunomodulatory strategies.

It should be noted that this necroinflammatory phenotype, present in a subgroup of patients (mainly men), is initiated by an “unresolved” hypoxic process through the expression of VEGFA. Few aspects have been remarkable in the analysis of the clinical trials of BVZ(a monoclonal antibody that inhibits VEGFA) when analyzing patients’ cohorts without stratifying in any way.^46^ For this reason, we evaluated the BVZ efficacy according to patient stratification based on our previous results. We divided our own glioma cohort treated with BVZ into necroinflammed and non-necroinflammed tumors, and surprisingly we observed an increment in the overall survival in necroinflammed patients compared to the other group. These data have several therapeutic implications, as some patients might not require BVZ treatment, while others benefit from it. Clinical benefit in glioma patients remains questionable considering the results obtained in clinical trials^47^ which are the subject of much controversy. Until now, the main issue regarding BVZ treatment is the lack of biomarkers and tumor patterns to identify patients who may benefit from it,^48^ as it has been done for other tumors,^49^ and how we have addressed it here. In addition, doubts remain about the real role of BVZ in the treatment of gliomas, it is even postulated as a promising therapy for brain necrosis after radiotherapy.^50^ Our analysis revealed that BVZ only has an effect under inflammatory tumor conditions, and we showed with ex vivo patient-derived tumor fragments (PDTFs) experiment a significant decrease in the necroinflammation gene signature, established in this study, after BVZ treatment. This anti-inflammatory effect could be the cause of the improvement in overall survival in patients after BZV treatment. Moreover, these data propose that necroinflammation gene signature expression could be used as a predictor of BVZ benefit. This therapy could only generate an increase in survival by reducing inflammation, not a cure for the disease due to the lack of direct effect on the tumor cell. We have demonstrated that the necroinflammation process described in this work is responsible for tumor aggressiveness, but we cannot rule out that there are other inflammatory processes that may also be modulating it, where BVZ treatment could have no effect.

Future experiments with larger cohorts would be necessary to confirm these effects and observe differences in overall survival since although our cohort is very representative, it includes a small number of patients. Thus, our results could have important implications for several aspects of glioma research and clinical practice.

## Supplementary material

Supplementary material is available online at *Neuro-Oncology* (https://academic.oup.com/neuro-oncology).

noae033_suppl_Supplementary_Material
