# Supplementary material for: Sexual-biased necroinflammation is revealed as a predictor of bevacizumab benefit in glioblastoma
Source: Neuro Oncol. 2024 Feb 27;26(7):1213–27. doi: 10.1093/neuonc/noae033 (PMC11226871; doi:10.1093/neuonc/noae033)
Supplement: noae033_suppl_Supplementary_Material [file noae033_suppl_supplementary_material.docx]

|  |  |  |  |  |  |  |
| --- | --- | --- | --- | --- | --- | --- |
|  | **Cancer** |  |  |  |  |  |
| **Cancer** | **abbreviation** | **Sex** | **n** | **Log-Rank** | ***p-value*** |  |
| Liver hepatocellular carcinoma | LIHC | Male | 249 | 1.182 | 0.277 |  |
|  |  | Female | 120 |  |  |  |
| Colon adenocarcinoma | COAD | Male | 156 | 2.721 | 0.099 |  |
|  |  | Female | 129 |  |  |  |
| Kidney renal clear cell carcinoma | KIRC | Male | 346 | 0.053 | 0.818 |  |
|  |  | Female | 188 |  |  |  |
| Stomach adenocarcinoma | STAD | Male | 263 | 1.360 | 0.243 |  |
|  |  | Female | 146 |  |  |  |
| Cholangiocarcinoma | CHOL | Male | 16 | 0.473 | 0.492 |  |
|  |  | Female | 20 |  |  |  |
| Kidney Chromophobe | KICH | Male | 38 | 0.396 | 0.529 |  |
|  |  | Female | 27 |  |  |  |
| Lung squamous cell carcinoma | LUSC | Male | 366 | 1.196 | 0.274 |  |
|  |  | Female | 129 |  |  |  |
| Thymoma | THYM | Male | 63 | 0.513 | 0.474 |  |
|  |  | Female | 56 |  |  |  |
| Rectum adenocarcinoma | READ | Male | 52 | 0.972 | 0.324 |  |
|  |  | Female | 42 |  |  |  |
| Lung adenocarcinoma | LUAD | Male | 240 | 0.134 | 0.714 |  |
|  |  | Female | 277 |  |  |  |
| Kidney renal papillary cell carcinoma | KIRP | Male | 213 | 1.944 | 0.163 |  |
|  |  | Female | 77 |  |  |  |
| Esophageal carcinoma | ESCA | Male | 159 | 3.343 | 0.067 |  |
|  |  | Female | 26 |  |  |  |
| **Glioblastoma multiforme** | **GBM** | Male | 367 | **4.61** | **0.031** |  |
|  |  | Female | 234 |  |  |  |
| **Head and Neck squamous cell carcinoma** | **HNSC** | Male | 385 | **5.522** | **0.022** |  |
|  |  | Female | 137 |  |  |  |
|  |  |  |  |  |  |  |
| Bladder Urothelial Carcinoma | BLCA | Male | 300 | 0.479 | 0.489 |  |
|  |  | Female | 106 |  |  |  |
| Skin Cutaneous Melanoma | SKCM | Male | 284 | 1.384 | 0.239 |  |
|  |  | Female | 172 |  |  |  |
| Pancreatic adenocarcinoma | PAAD | Male | 99 | 0.997 | 0.318 |  |
|  |  | Female | 80 |  |  |  |
| Sarcoma | SARC | Male | 120 | 0.393 | 0.531 |  |
|  |  | Female | 145 |  |  |  |
| Thyroid carcinoma | THCA | Male | 136 | 1.851 | 0.174 |  |
|  |  | Female | 371 |  |  |  |
| Low grade glioma | LGG | Male | 291 | 0.575 | 0.448 |  |
|  |  | Female | 237 |  |  |  |
|  |  |  |  |  |  |  |

**Supplementary Table 1. Sex disparities in Pan-Cancer survival rates.** Male and Female overall survival rates comparison in different cancer types using the Log-rank test (Mantel-Cox), data was extracted from TCGA PAN-Cancer cohort.

| **Glioma cohort** | |  |  | **BVZ GBM cohort** | |  |
| --- | --- | --- | --- | --- | --- | --- |
| **Patient characteristics** | | n=104 |  | **Patient characteristics** | | n=36 |
| **Median age (years)** | | 56 |  | **Median age (years)** | | 59 |
| **Median KPS(range)** | | 90(70-100) |  | **Median KPS(range)** | | 90(70-100) |
| **Sex** |  |  |  | **Sex** |  |  |
| Female |  | 37(35,6%) |  | Female |  | 17 (47,2%) |
| Male |  | 67 (64,4%) |  | Male |  | 19 (52,8%) |
| **Race** |  |  |  | **Race** |  |  |
| Black or African American | | 0 |  | Black or African American | | 0 |
| White |  | 104(100%) |  | White |  | 36 (100%) |
| **Extent of resection** | |  |  | **Extent of resection** | |  |
| Subtotal resection | | 39(37,4%) |  | Subtotal resection | | 11 (30,6%) |
| Total resection | | 65(62,6)% |  | Total resection | | 25 (69,4%) |
| **Histological Type** | |  |  | **Histological Type** | |  |
| Astrocytoma | | 104(100%) |  | Astrocytoma | | 36 (100%) |
| Oligodendroglioma | | 0 |  | Oligodendroglioma | | 0 |
| **Grade** |  |  |  | **Grade** |  |  |
| Astrocytome IDHmut (2-4) | | 31(29,8%) |  | Astrocytome IDHmut (2-4) | | 0 |
| GBM (IDHwt 4) | | 73(70,2%) |  | GBM (IDHwt 4) | | 36(100%) |
| **Molecular status** | |  |  | **Molecular status** | |  |
| IDH mutated | | 31 (29,8%) |  | IDH mutated | | 0 (0%) |
| MGMT methylated | | 39 (37,5%) |  | MGMT methylated | | 10(27,8) |
| **Treatment** |  |  |  |  |  |  |
| Stupp protocol | | 90(86,8%) |  |  |  |  |

**Supplementary Table 2. Summary of clinical features from our Glioma (n=104) and BVZ cohorts (n=35).**

** Supplementary Figure S1. Male glioma patients have lower survival rates than female patients.** (**A-C**) Kaplan-Meier overall survival curves of patients from the glioma cohort (TCGA LGG+GBM) (n = 1135) (**A**). Astrocytoma IDHmut cohort (TCGA LGG+GBM selecting IDH mut) (n = 563) (**B**) and GBM cohort (n = 561) (TCGA GBM IDH wt) (**C**). The patients in each cohort were stratified by sex into two groups (female and male). (**D**) Karnofsky’s performance status for males and females in the overall GBM patient population. (**E-F**) Frequency of somatic nonsilent mutations in Glioma cohort (TCGA GBM+LGG) (**E**) and GBM cohort (TCGA GBM) (**F**) classified according to sex. The data are shown as means ± SEM. The significance of gene expression was analyzed using a Student's t-test. Kaplan-Meier curves were compared with the log-rank test (Mantel-Cox). *P ≤ 0.05; **P ≤ 0.01; ***P ≤ 0.001, ****P≤0.0001. n.s., not significant.


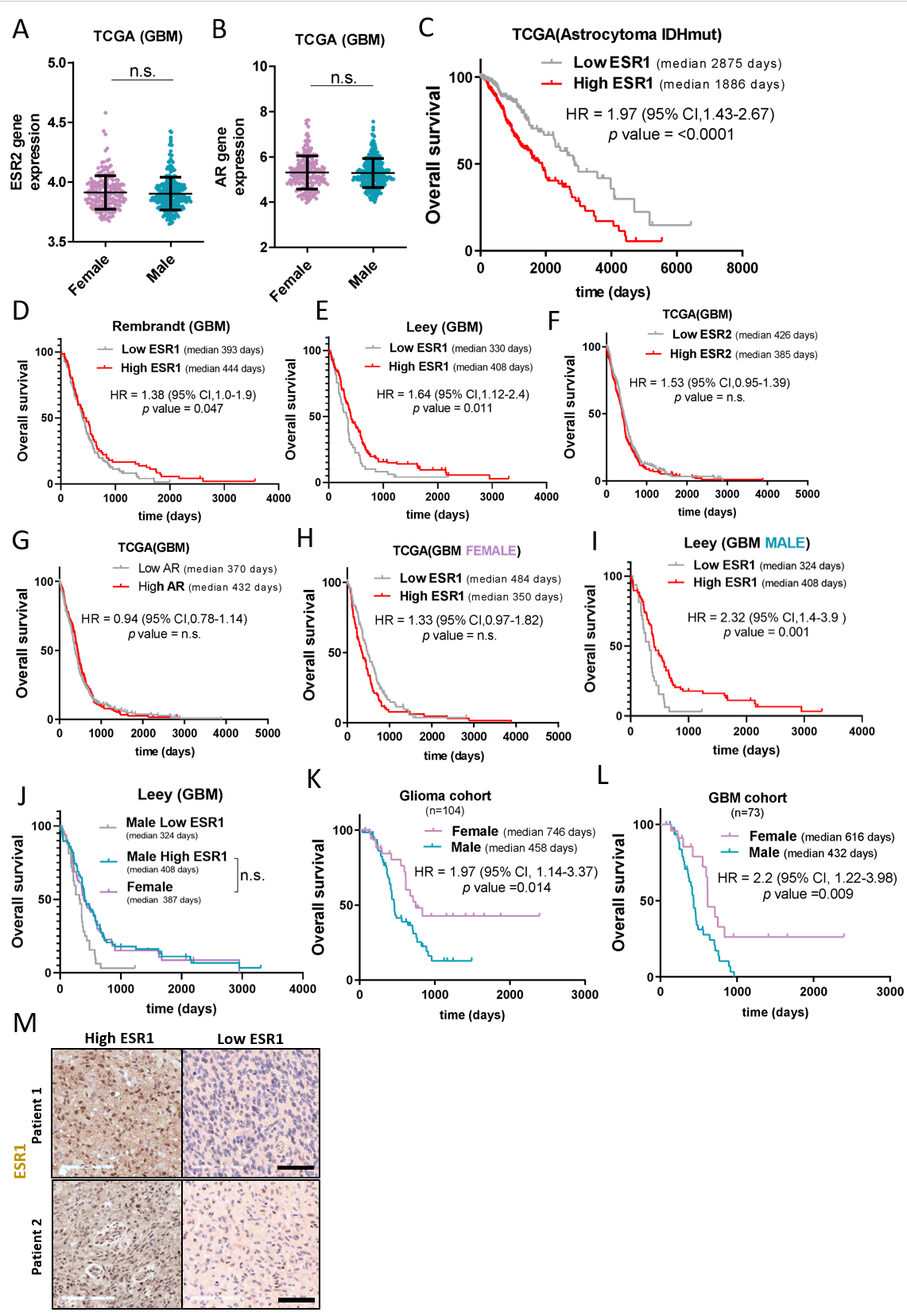


**Supplementary Figure S2. Involvement of the expression of hormone receptors; ESR1, ESR2, and AR in GBM tumor aggressiveness according to sex.** (**A-B**) ESR2 (**A**) and AR (**B**) expression analysis by RNA-Seq in the GBM cohort (TCGA GBM IDH wt) grouped according to sex. (**C**) Kaplan-Meier overall survival curves of patients from astrocytoma IDH mut cohort (TCGA LGG+GBM IDH mut) (n = 559) stratified according to their ESR1 gene expression measured by RNA-Seq. **(D-E)** Kaplan-Meier overall survival curves of patients with GBM from Rembrandt (n=173) (D) and Leey (n=180) (E) dataset stratified according to their ESR1 gene expression **(F-G)** Kaplan-Meier overall survival curves of patients from the GBM cohort (n = 518) (TCGA GBM IDH wt) stratified according to high/low ESR2 (**F**) and AR (**G**) expression. (**H)** Kaplan-Meier overall survival curves of GBM female patients (TCGA GBM IDH wt cohort) according to ESR1 gene expression measured by RNA-Seq analysis. **(I)** Kaplan-Meier overall survival curves of male GBM patients from Leey dataset (n=110). **(J)** Kaplan-Meier overall survival curve from GBM cohort (Leey) (n =180) stratified in three subgroups male high ESR1, male low ESR1, and female.(**K-L**) Kaplan-Meier overall survival curves of patients stratified based on sex from our own glioma (n = 104) (**K**) and GBM cohort (n= 73) (**L**). (**M**) Representative IHC image of high ESR1 and low ESR1 in GBM tumor sections from our glioma cohort. The data are shown as means ± SEM. The significance of gene expression was analyzed using Student's t-test. Kaplan-Meier curves were compared with the log-rank test (Mantel-Cox). *P ≤ 0.05; **P ≤ 0.01; ***P ≤ 0.001, ****P≤0.0001. n.s., not significant.

**Supplementary Figure S3. Establishment of necrotic phenotype associated with tumors with low ESR1 expression.** (**A**) Volcano plot showing differential expression in GBM comparing necrotic zones with different areas of the tumor [leading edge (LT), infiltrating tumor cells (IT), celullar tumor (CT), and vascular zone (MVP)], using IvyGap dataset. (**B**) Gene ontology analysis (DAVID Gene Ontology program) of biological processes based on 40 genes up-regulated in high necrotic tumors compared to low necrotic tumors in our own GBM cohort (data obtained by RNA-seq analysis). (**C-D**) Single-cell RNA-Seq analysis of S100A9 expression in human GBM samples grouped by clustering. Single-cell RNA-Seq was obtained from the Single Cell Portal (GSE182109). (**E**) Dot plot expression obtained by scRNA-Seq of CD68, S100A9, TREM1, CLEC5A and MS4A4A genes in human GBM samples grouped by cluster. The color scale indicates the average expression of selected genes and the circle sizes indicate the proportion of cells expressing the selected gene. Single-cell RNA-Seq was obtained from the Single Cell Portal (GSE182109). (**F**) Establishment of necroinflammation gene expression signature and representative Venn Diagram showing genes co-expressed with S100A9 and genes expressed in necrotic zones.(**G**) Heatmap showing common genes co-expressing with S100A9 (TCGA GBM IDH wt cohort) that are present exclusively in necrotic areas of the tumor using the IvyGap dataset.

**
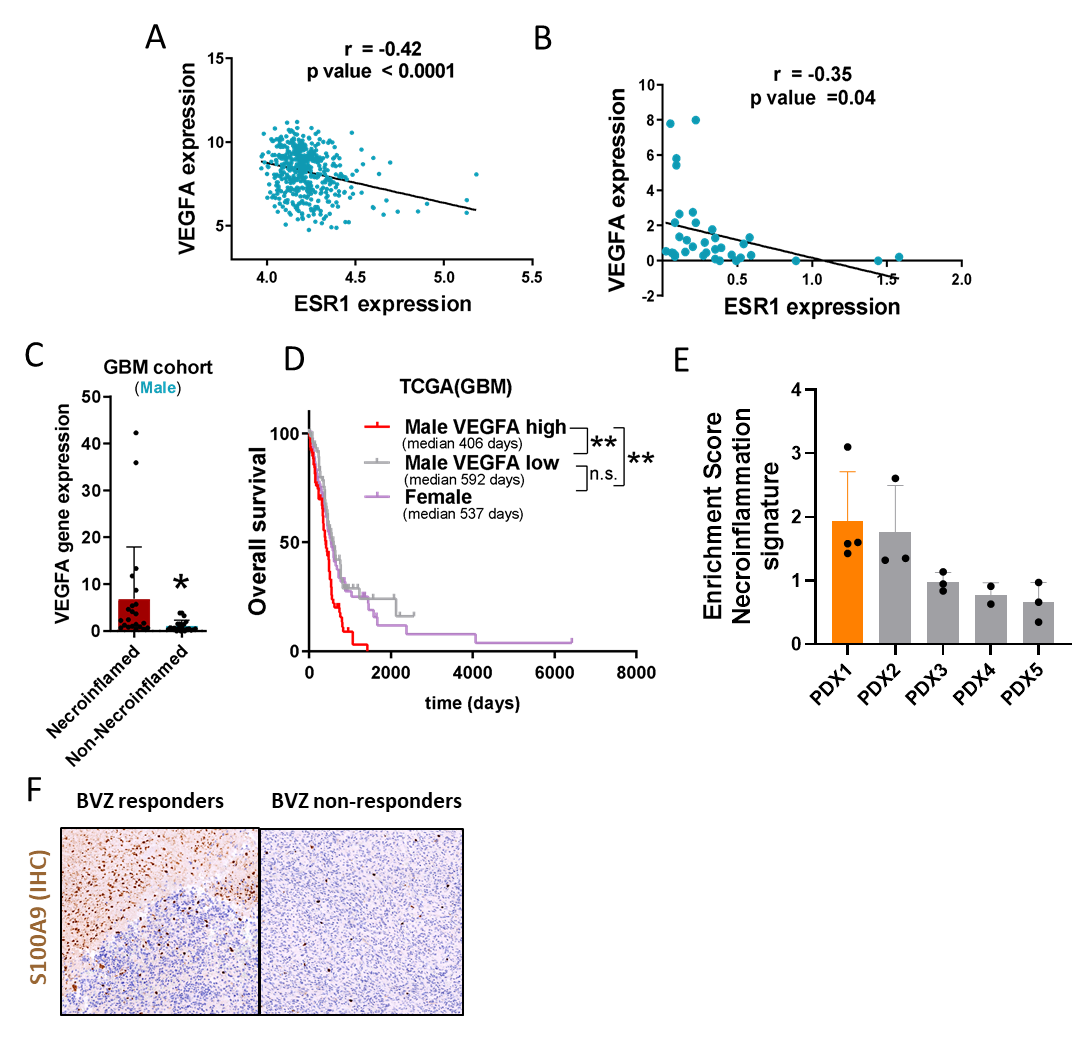
**

**Supplementary Figure S4**. **The expression of VEGFA in GBM correlates with tumor aggressiveness in male patients.** (**A-B**) Correlation between VEGFA and ESR1 expression in the GBM cohort (TCGA GBM IDH wt) (n = 481) (**A**) and in our own glioma cohort (n =35) (**B**), measured by RNA-seq and qRT-PCR respectively. (**C**) Kaplan-Meier overall survival curves of patients from the GBM cohort (TCGA GBM IDH wt) (n =256). The patients are stratified into three subgroups, high VEGFA male, low VEGFA male, and female. (**D**) Quantification of VEGFA gene expression measured by qRT-PCR in male patients from our own Glioma cohort (n= 35) stratified into necroinflamed and non-necroinflamed tumors. (**E**) Quantification of necroinflammation gene signature expression in different PDX experiments. (**F**) Representative images of S100A9 IHC staining in sections from PDTFs samples stratified into possible BVZ responders and possible BVZ non-responders. The data are represented with means ± SEM. A comparison of Kaplan-Meier curves was performed with a log-rank test (Mantel-Cox). Correlation was performed with the Pearson correlation coefficient. *P ≤ 0.05; **P ≤ 0.01; ***P ≤ 0.001, ****P≤0.0001. n.s., not significant.

**Supplementary Table 3. qRT-PCR primers**

| **gene** | **Forward (5´-3´)** | **Reverse (3´-5´)** |
| --- | --- | --- |
| **ADAM8** | TGCTGGAGGTGGTGAATCACGT | TCAGGAGGTTCTCCAGTGTGAC |
| **ADM** | GACATGAAGGGTGCCTCTCGAA | CCTGGAAGTTGTTCATGCTCTGG |
| **AKAP12** | AGAAAGGAGCCCTGAACGGTCA | CCGCTGACTTAGTAGCCATCTC |
| **ALOX5AP** | AAGTGGAGCACGAAAGCAGGAC | AGACCAGAGCACAGCGAGGAAA |
| **ARL4C** | AGGTGACCAAGTTCGCCGAGAA | GCTGCTTCTCAATCTCTGCCAC |
| **C5AR1** | ATCTGGTGCCAGAACTTCCGAG | CTGTAGTCCACGCCACACAACA |
| **CCL2** | AGAATCACCAGCAGCAAGTGTCC | TCCTGAACCCACTTCTGCTTGG |
| **CCL20** | AAGTTGTCTGTGTGCGCAAATCC | CCATTCCAGAAAAGCCACAGTTTT |
| **CCL3** | ACTTTGAGACGAGCAGCCAGTG | TTTCTGGACCCACTCCTCACTG |
| **CCL7** | ACAGAAGGACCACCAGTAGCCA | GGTGCTTCATAAAGTCCTGGACC |
| **CHI3L1** | CCACAGTCCATAGAATCCTCGG | TGCCTGTCCTTCAGGTACTGCA |
| **CLEC5A** | TTGTCAACACGCCAGAGAAACTG | CAACGCCACCTTTTCTCTTCACG |
| **ERα** | GCTTACTGACCAACCTGGCAGA | GGATCTCTAGCCAGGCACATTC |
| **FCGR2B** | TCCAAGCCTGTGACCATCACTG | CCACTACAGCAGCAACAATGGC |
| **GAPDH** | GTCTCCTCTGACTTCAACAGCG | ACCACCCTGTTGCTGTAGCCAA |
| **GBE1** | GCCTTGACTTACCTCATGTTGGC | AGCACAGAGCTGGCATTCCTGA |
| **HILPDA** | TGATGGAGTCCCTAGAGGGCTT | GCCAGTATGGAAGGAGGTCTTAT |
| **HMOX1** | CCAGGCAGAGAATGCTGAGTTC | AAGACTGGGCTCTCCTTGTTGC |
| **HSPA6** | GCTGAGCAAGATGAAGGAGACG | GATGATCCGCAACACGTTGAGC |
| **IGFBP5** | CGTGCTGTGTACCTGCCCAATT | ACTTGTCCACGCACCAGCAGAT |
| **IL1B** | CCACAGACCTTCCAGGAGAATG | GTGCAGTTCAGTGATCGTACAGG |
| **IL1RAP** | CTGAGGATCTCAAGCGCAGCTA | AGCAGGACTGTGGCTCCAAAAC |
| **IL6** | AGACAGCCACTCACCTCTTCAG | TTCTGCCAGTGCCTCTTTGCTG |
| **LDHA** | GGATCTCCAACATGGCAGCCTT | AGACGGCTTTCTCCCTCTTGCT |
| **LIF** | AGATCAGGAGCCAACTGGCACA | GCCACATAGCTTGTCCAGGTTG |
| **PLOD2** | GACAGCGTTCTCTTCGTCCTCA | CTCCAGCCTTTTCGTGGTGACT |
| **SLC2A3** | TGCCTTTGGCACTCTCAACCAG | GCCATAGCTCTTCAGACCCAAG |
| **SLC39A14** | CTGGACCACATGATTCCTCAGC | AGAGTAGCGGACACCTTTCAGC |
| **TSLP** | TATCTGGTGCCCAGGCTATTCG | TGAAGCGACGCCACAATCCTTG |
| **VEGFA** | TTGCCTTGCTGCTCTACCTCCA | GATGGCAGTAGCTGCGCTGATA |
| **ZNF395** | CCTTTCCTGCTGGACGAACCAG | ATGGAGGGCTTTGACGTGTCGT |

**Supplementary Table 4. Genes up regulated in necrotic zones compared to non-necrotic zones.**

| **Up-Regulated Genes** | **Spearman's Correlation** | **p-Value** | **Up-Regulated Genes2** | **Spearman's Correlation3** | **p-Value4** |
| --- | --- | --- | --- | --- | --- |
| **IL8** | 55.700 | 2,88E-29 | **CLEC2B** | 2.881 | 2,91E-11 |
| **VEGFA** | 21.440 | 1,05E-66 | **RGS1** | 2.880 | 2,12E-11 |
| **HILPDA** | 17.109 | 1,82E-55 | **SYTL2** | 2.878 | 1,01E-30 |
| **CCL20** | 15.142 | 5,90E-18 | **JUN** | 2.871 | 8,66E-31 |
| **PI3** | 14.493 | 8,47E-13 | **IL1A** | 2.868 | 1,59E-13 |
| **ADM** | 13.935 | 5,33E-42 | **CSF3** | 2.860 | 1,35E-07 |
| **CA9** | 12.904 | 3,88E-51 | **GAPDH** | 2.858 | 1,35E-21 |
| **NDRG1** | 12.779 | 3,64E-47 | **C4orf47** | 2.846 | 2,00E-25 |
| **TREM1** | 12.498 | 1,30E-25 | **CEBPB** | 2.841 | 2,52E-15 |
| **PTX3** | 10.741 | 3,10E-18 | **PLP2** | 2.835 | 1,06E-20 |
| **CA12** | 10.487 | 5,45E-42 | **KDM3A** | 2.819 | 2,13E-27 |
| **ANGPTL4** | 10.431 | 4,71E-34 | **FAM115C** | 2.819 | 1,06E-40 |
| **LOC100509105** | 10.212 | 1,65E-24 | **ISG20** | 2.816 | 2,50E-20 |
| **C15orf48** | 9.754 | 3,21E-28 | **BNIP3L** | 2.812 | 1,17E-35 |
| **CHI3L1** | 9.505 | 3,66E-17 | **MET** | 2.774 | 7,58E-11 |
| **BIRC3** | 8.872 | 8,71E-15 | **DNAJB1** | 2.762 | 9,46E-28 |
| **SLPI** | 8.740 | 2,74E-12 | **HOXA4** | 2.757 | 7,41E-09 |
| **LOX** | 8.691 | 1,64E-30 | **TIMP1** | 2.756 | 2,01E-07 |
| **SLAMF9** | 8.449 | 4,77E-22 | **HAS2** | 2.743 | 4,17E-16 |
| **PTGS2** | 8.142 | 1,34E-16 | **METTL21B** | 2.732 | 1,08E-08 |
| **MT1H** | 8.063 | 1,00E-22 | **LIPG** | 2.711 | 1,74E-14 |
| **PLIN2** | 8.013 | 4,57E-35 | **CXCL5** | 2.702 | 2,13E-05 |
| **CXCL3** | 7.614 | 3,50E-13 | **SLC15A4** | 2.693 | 1,76E-32 |
| **SERPINE1** | 7.572 | 1,49E-25 | **RARRES2** | 2.693 | 6,26E-06 |
| **HK2** | 7.556 | 3,65E-45 | **BEND5** | 2.692 | 2,24E-18 |
| **C4orf6** | 7.531 | 4,80E-22 | **TNFRSF12A** | 2.687 | 7,87E-13 |
| **SAA1** | 7.463 | 1,03E-07 | **MYC** | 2.673 | 2,64E-21 |
| **MT1X** | 7.422 | 3,21E-42 | **FKBP9L** | 2.666 | 9,00E-09 |
| **ZNF395** | 7.210 | 3,73E-61 | **MKNK2** | 2.665 | 4,21E-18 |
| **SPAG4** | 7.078 | 3,07E-47 | **IER3** | 2.662 | 1,83E-08 |
| **ANKRD37** | 7.063 | 2,04E-29 | **GFPT2** | 2.659 | 1,13E-14 |
| **SLC39A14** | 6.747 | 3,45E-36 | **EGR1** | 2.656 | 5,76E-15 |
| **SLC2A3** | 6.677 | 1,38E-36 | **CSDA** | 2.650 | 9,75E-21 |
| **SLC2A1** | 6.565 | 6,27E-36 | **SLC16A10** | 2.650 | 2,78E-17 |
| **CAV1** | 6.285 | 9,15E-27 | **DDIT4** | 2.647 | 1,59E-16 |
| **EGLN3** | 6.148 | 1,27E-31 | **GLUL** | 2.631 | 5,78E-17 |
| **ERO1L** | 5.926 | 1,49E-39 | **SEC61G** | 2.620 | 1,83E-06 |
| **PLOD2** | 5.781 | 7,08E-44 | **RGS17** | 2.618 | 2,53E-21 |
| **TMEM45A** | 5.744 | 1,41E-33 | **JMJD6** | 2.612 | 1,31E-29 |
| **ANG** | 5.689 | 1,25E-35 | **TFPI2** | 2.607 | 2,26E-05 |
| **ATF3** | 5.687 | 1,82E-37 | **GRB10** | 2.606 | 3,70E-11 |
| **AKAP12** | 5.659 | 3,73E-27 | **KRT75** | 2.597 | 8,44E-12 |
| **IL1B** | 5.624 | 2,82E-16 | **S100A8** | 2.593 | 3,86E-06 |
| **GPRC5A** | 5.614 | 9,69E-33 | **GADD45A** | 2.575 | 1,21E-15 |
| **HMOX1** | 5.604 | 3,60E-23 | **FOS** | 2.555 | 3,42E-15 |
| **NAMPT** | 5.561 | 4,94E-31 | **CXCR4** | 2.546 | 5,08E-13 |
| **MT1G** | 5.469 | 3,81E-21 | **STC2** | 2.545 | 7,57E-20 |
| **NRN1** | 5.461 | 2,54E-34 | **STBD1** | 2.538 | 2,17E-20 |
| **DDIT3** | 5.342 | 3,83E-37 | **SLCO4A1** | 2.536 | 2,99E-19 |
| **ICAM1** | 5.278 | 1,71E-19 | **KCNE4** | 2.533 | 4,06E-17 |
| **IL6** | 5.274 | 6,03E-10 | **CLCF1** | 2.531 | 8,41E-13 |
| **BCL2A1** | 5.268 | 4,73E-21 | **EMP3** | 2.526 | 1,05E-15 |
| **HSPA6** | 5.165 | 1,16E-22 | **ATP13A3** | 2.521 | 2,13E-31 |
| **ARRDC3** | 5.117 | 2,05E-43 | **H19** | 2.520 | 2,99E-04 |
| **CHI3L2** | 5.072 | 1,20E-08 | **RGS2** | 2.516 | 2,04E-09 |
| **LOC154761** | 5.044 | 1,86E-36 | **P4HA2** | 2.516 | 1,19E-22 |
| **LGALS3** | 5.035 | 1,37E-16 | **TPI1** | 2.508 | 7,79E-28 |
| **MMP7** | 4.992 | 1,19E-08 | **PCSK1** | 2.505 | 1,56E-10 |
| **PLA2G2A** | 4.956 | 7,25E-08 | **FBLN5** | 2.498 | 2,04E-08 |
| **IGFBP5** | 4.945 | 1,90E-26 | **C1orf51** | 2.498 | 2,60E-16 |
| **CXCL2** | 4.905 | 1,16E-10 | **C8orf22** | 2.494 | 9,19E-10 |
| **C5orf62** | 4.873 | 5,25E-38 | **PPP1R3B** | 2.488 | 4,40E-30 |
| **SLN** | 4.809 | 4,25E-09 | **RAB20** | 2.477 | 7,49E-22 |
| **ENO2** | 4.798 | 2,81E-35 | **TGFBI** | 2.467 | 3,47E-09 |
| **PFKFB4** | 4.761 | 5,29E-47 | **LOC100506498** | 2.466 | 1,21E-13 |
| **PPP1R15A** | 4.731 | 3,49E-31 | **RP9P** | 2.466 | 2,63E-14 |
| **RNASE4** | 4.693 | 8,29E-32 | **ALDOA** | 2.464 | 3,61E-22 |
| **MT2A** | 4.683 | 1,04E-20 | **CLEC5A** | 2.463 | 2,85E-15 |
| **LOC100505994** | 4.592 | 2,55E-23 | **TSLP** | 2.463 | 1,47E-13 |
| **PDK1** | 4.577 | 1,24E-30 | **IL1RAP** | 2.453 | 1,35E-13 |
| **MIR210HG** | 4.565 | 4,15E-41 | **SMS** | 2.450 | 4,55E-35 |
| **SAA2** | 4.523 | 1,66E-06 | **CYTIP** | 2.447 | 4,44E-15 |
| **LOC100653010** | 4.506 | 3,84E-19 | **RRAGD** | 2.440 | 2,44E-25 |
| **C8orf4** | 4.467 | 2,09E-16 | **DUSP1** | 2.430 | 9,78E-13 |
| **GBE1** | 4.453 | 8,99E-45 | **FAM110C** | 2.427 | 4,20E-08 |
| **PDPN** | 4.406 | 4,03E-18 | **GPX3** | 2.424 | 1,76E-06 |
| **RDH10** | 4.378 | 1,28E-23 | **WSB1** | 2.422 | 1,10E-23 |
| **NFKBIZ** | 4.339 | 7,17E-14 | **GJB2** | 2.415 | 8,21E-08 |
| **CCL7** | 4.333 | 1,95E-09 | **MXD1** | 2.411 | 1,48E-28 |
| **FAM180A** | 4.326 | 1,02E-11 | **AQP9** | 2.405 | 8,70E-11 |
| **SPINK1** | 4.319 | 1,22E-15 | **HSPA5** | 2.403 | 5,63E-25 |
| **BNIP3** | 4.309 | 2,27E-32 | **FRMD3** | 2.401 | 1,16E-19 |
| **IGFBP3** | 4.284 | 3,05E-08 | **FGF11** | 2.397 | 3,05E-19 |
| **SLC6A6** | 4.232 | 2,20E-29 | **ID2** | 2.384 | 2,89E-16 |
| **INSIG2** | 4.219 | 2,56E-42 | **CCL3** | 2.383 | 4,03E-07 |
| **NNMT** | 4.207 | 9,12E-11 | **NFKBIA** | 2.380 | 2,01E-11 |
| **MAFF** | 4.192 | 2,77E-36 | **LOC100653017** | 2.374 | 6,30E-13 |
| **ARL4C** | 4.184 | 1,54E-45 | **MMP19** | 2.370 | 3,24E-13 |
| **SOD2** | 4.176 | 3,93E-12 | **C5orf46** | 2.361 | 1,26E-08 |
| **LOC100652805** | 4.146 | 1,04E-41 | **PIM1** | 2.360 | 2,45E-15 |
| **AK4** | 4.055 | 1,83E-39 | **ADAM8** | 2.358 | 1,67E-16 |
| **TRIB3** | 4.005 | 1,23E-22 | **SLC6A8** | 2.357 | 1,09E-19 |
| **CEBPD** | 3.963 | 4,87E-25 | **SESN2** | 2.356 | 8,47E-17 |
| **SDC4** | 3.919 | 7,35E-18 | **SLC3A2** | 2.355 | 2,02E-17 |
| **LIF** | 3.878 | 6,37E-13 | **WTAP** | 2.354 | 1,62E-15 |
| **S100A10** | 3.874 | 1,19E-22 | **ALOX5AP** | 2.336 | 1,35E-07 |
| **PYGL** | 3.799 | 3,28E-28 | **TNIP1** | 2.330 | 2,72E-21 |
| **PGK1** | 3.726 | 3,32E-34 | **EMP1** | 2.326 | 2,69E-16 |
| **SOCS3** | 3.643 | 2,14E-21 | **DHRS3** | 2.325 | 3,03E-14 |
| **CHRDL2** | 3.636 | 5,58E-20 | **ENO1** | 2.321 | 2,89E-13 |
| **SNHG12** | 3.633 | 6,46E-19 | **MIR100HG** | 2.319 | 4,50E-16 |
| **NOL3** | 3.626 | 3,85E-38 | **CCDC109B** | 2.318 | 1,01E-16 |
| **CRABP2** | 3.606 | 7,14E-20 | **PTGES** | 2.317 | 2,76E-11 |
| **BHLHE40** | 3.601 | 5,80E-26 | **SYNPO** | 2.315 | 3,49E-14 |
| **HSPA1B** | 3.584 | 1,20E-28 | **JUNB** | 2.299 | 7,31E-12 |
| **NUPR1** | 3.559 | 2,63E-23 | **UBC** | 2.299 | 1,34E-21 |
| **TMEM158** | 3.559 | 1,92E-23 | **MCHR1** | 2.294 | 1,13E-20 |
| **CCL2** | 3.519 | 2,73E-06 | **OSGIN1** | 2.291 | 1,01E-18 |
| **PLAUR** | 3.472 | 8,99E-15 | **ELL2** | 2.288 | 7,51E-19 |
| **DDIT4L** | 3.398 | 1,85E-14 | **KLF5** | 2.284 | 5,39E-12 |
| **CHRNA9** | 3.389 | 5,78E-13 | **MTMR11** | 2.282 | 9,99E-21 |
| **C10orf10** | 3.370 | 6,09E-14 | **C1R** | 2.282 | 4,98E-08 |
| **PCOLCE2** | 3.316 | 5,23E-14 | **TMEM38B** | 2.281 | 1,02E-17 |
| **CYR61** | 3.285 | 4,20E-17 | **VKORC1** | 2.281 | 5,48E-28 |
| **TNC** | 3.270 | 3,03E-24 | **MYADM** | 2.278 | 4,04E-17 |
| **SPOCD1** | 3.268 | 4,94E-20 | **RGS16** | 2.270 | 2,06E-08 |
| **IGFBP2** | 3.262 | 4,06E-20 | **ADSSL1** | 2.269 | 2,33E-15 |
| **CD44** | 3.256 | 5,18E-23 | **PPP1R3C** | 2.269 | 1,42E-14 |
| **GADD45B** | 3.229 | 1,72E-20 | **C1QTNF1** | 2.264 | 2,73E-08 |
| **NFIL3** | 3.214 | 4,87E-31 | **CHPF** | 2.263 | 2,13E-14 |
| **S100A9** | 3.212 | 4,26E-10 | **NGLY1** | 2.262 | 9,48E-26 |
| **FCGR2B** | 3.209 | 5,45E-12 | **SERTAD1** | 2.262 | 5,88E-16 |
| **P4HA1** | 3.188 | 5,30E-39 | **ATF5** | 2.252 | 4,43E-15 |
| **GBP2** | 3.169 | 1,36E-16 | **SNHG1** | 2.248 | 6,47E-12 |
| **IL1RN** | 3.161 | 5,70E-17 | **TAGLN** | 2.248 | 2,58E-05 |
| **G0S2** | 3.160 | 9,11E-11 | **NT5C3** | 2.240 | 4,60E-14 |
| **ABCA1** | 3.158 | 1,69E-30 | **MIF** | 2.235 | 8,17E-11 |
| **FAM20C** | 3.157 | 9,41E-23 | **FTH1** | 2.234 | 1,59E-12 |
| **EIF4EBP1** | 3.143 | 8,13E-23 | **SLC25A37** | 2.223 | 2,45E-14 |
| **DOK5** | 3.141 | 2,84E-24 | **CNN1** | 2.221 | 1,27E-07 |
| **LOC100505584** | 3.132 | 3,06E-16 | **RAB42** | 2.220 | 2,07E-14 |
| **TNFAIP2** | 3.116 | 1,75E-07 | **CTAGE6P** | 2.220 | 7,54E-17 |
| **MIR155HG** | 3.111 | 9,96E-13 | **ANXA2** | 2.219 | 4,39E-12 |
| **DNAJB9** | 3.092 | 3,38E-19 | **FOSL1** | 2.214 | 7,99E-12 |
| **LOC100507165** | 3.091 | 2,65E-15 | **CD300A** | 2.212 | 5,18E-10 |
| **LOC100652898** | 3.063 | 2,64E-15 | **HMGA1** | 2.211 | 5,15E-14 |
| **EPHA1-AS1** | 3.061 | 1,18E-17 | **SCG2** | 2.209 | 4,51E-08 |
| **SLC16A1** | 3.058 | 2,11E-35 | **SIAH2** | 2.207 | 2,54E-25 |
| **SPP1** | 3.047 | 2,76E-14 | **MSR1** | 2.206 | 4,56E-08 |
| **LDHA** | 3.046 | 1,68E-44 | **C5AR1** | 2.206 | 7,21E-12 |
| **RND3** | 3.012 | 1,78E-17 | **EFEMP1** | 2.203 | 4,09E-09 |
| **CP** | 2.994 | 4,91E-08 | **IRS2** | 2.201 | 1,11E-12 |
| **EGR2** | 2.988 | 9,77E-16 | **C20orf111** | 2.199 | 1,77E-16 |
| **TNFAIP3** | 2.982 | 6,81E-11 | **CDCP1** | 2.199 | 1,18E-15 |
| **GLIPR1** | 2.958 | 1,01E-17 | **VIM** | 2.197 | 8,29E-20 |
| **GPNMB** | 2.952 | 7,64E-16 | **CHD1L** | 2.196 | 2,21E-22 |
| **LOC100652886** | 2.946 | 1,58E-09 | **LTF** | 2.193 | 1,21E-02 |
| **EFCAB3** | 2.939 | 1,55E-24 | **ITGA3** | 2.193 | 3,65E-11 |
| **UPP1** | 2.925 | 1,74E-14 | **STC1** | 2.193 | 6,50E-06 |
| **WEE1** | 2.902 | 8,29E-20 | **PFKP** | 2.187 | 1,62E-19 |
| **FAM162A** | 2.901 | 7,72E-29 | **UAP1** | 2.185 | 4,03E-19 |

**Supplemental Material and Methods**

**Human Glioma Cells.**

The Human cells were derived from surgical specimens obtained from patients under treatment at "Hospital 12 de Octubre" in Madrid, Spain. The cell lines were previously characterized as IDH wilt type and named PDX1-5. These specimens were collected with the written consent of the patients and the approval of the Ethical Committee (CEI 14/023) and belong to the Biobank of the hospital. For maintenance cells culture were done with Complete Media (CM), which consisted of a media of Neurobasal supplemented with B27 (1:50) and GlutaMAX (1:100) from Thermo-Fisher-Scientific, along with penicillin-streptomycin (at a 1:100 ratio) from Lonza. Additionally, 0.4% heparin from Sigma-Aldrich was included in the medium, along with 40 ng/ml of EGF and 20 ng/ml of bFGF2 from Peprotech.

**RNAseq assay.**

*Differential expression analysis.* The Bioconductor edgeR package was used to import raw counts into R statistical software and calculate normalization factors to scale the raw library sizes using the TMM (weighted trimmed mean of M-values) method. Differential expression analysis was performed using the Bioconductor limma package and the voom transformation. To improve the statistical power of the analysis, only genes expressed in at least one sample (CPM >= 0.1) were considered. A qval threshold of <= 0.05 and a minimum fold change of 1.2 were used to define differentially expressed genes*.*

**Quantification of the Immunohistochemical (IHC).**

Density measurements of vascular density, necrotic area, pericytes coverage, collagen type IV+ basement membrane coverage, and distribution of endothelial junctional molecule VE-cadherin were performed with ImageJ software (http:// rsb.info.nih.gov/ij). Furthermore, in the case of the necrotic area, we used a score to grade the intensity of the quantified necrosis. To calculate vasculature per random field areas was measured in the intratumoral regions of tumor sections.

**Bioinformatics studies.**

The Cancer Genome Atlas (TCGA) Pan-Cancer (PANCAN), GBM, LGG, and GBM+LGG dataset was accessed via cBioPortal (https://www.cbioportal.org/), UCSC xena-browser (https://xenabrowser.net), and Gliovis (http:// gliovis.bioinfo.cnio.es) for extraction of the data: overall survival, gene’s expression level and the distribution of the different genetic alterations. Kaplan–Meier survival curves were done upon stratification based on low and high groups using expression values from each gene. First, we selected a cluster of at least 500 genes co-expressed with the corresponding gene (ESR1 or S100A9), using the highest value of the Spearman correlation. Then, the “David gene ontology” analysis associates the expression of these genes with the biological processes involved. The hypoxic-related genes signature included hypoxia and VEGFA pathways genes. IvyGap date set analysis (http://glioblastoma.alleninstitute.org/) was used to analyze gene signature enrichment between the different anatomic structures identified in the tumor (Supplementary Table 4). With this, we established both, hypoxia and necroinflammation gene signatures. Gene Set Enrichment Analysis (GSEA) pre-ranked was computed into the TCGA GBM cohort (RNAseq (IlluminaHiSeq)) using ESR1 gene co-expression and GSEA software (Gene Set Enrichment Analysis, RRID:SCR003199, v4.2.1) and Gene collections were obtained from MSigDB (v7.5.1).

**Patient-derived tumor fragment (PDTFs).**

PDTFs cultures from GBM patients were prepared by cutting 1mm sections of fresh tumor tissue, cultured in collagen with matrigel mixture, as described in ^14^, and incubated 24h with Bevacizumab (Zirabev, Pfizer) (10µg/ml) and isotype control (Bio- Legend, 401401) as control. PDTFs will be grown in media complete media: DMEM (Thermo-Fisher-Scientific) supplemented with FBS (10%); penicillin-streptomycin (1:100); EGF (40 ng/ml) and bFGF2 (20 ng/ml) (PeproTech). After that, we froze the PDTFs at -80ºC for subsequent analysis at the transcriptional level using qRT-PCR.

**List Immunofluorescence (IF) and Immunohistochemical (IHC) staining.**

The following primary antibodies: αSMA (1:100,#19245, Cell signaling),CD34(1:100,#END-L-CE, Leica), COLIV (1:100, MO785 ,Dako),ERα (1:50, #ab32063, abcam), S100A9 (1:800, #34425, Cell Signaling), VE-Cadherin (1:100,#2500,Cell Signaling), CD15(1:200, #4744S, Cell Signaling), CLECL5A(1:100, #15517802, Thermo Fisher), CD11b(1:300,#133357, Abcam), CD15(1:100,# 54192, Cell signaling),MCHII(1:500,# 97971, Cell Signaling) and VEGFA (1:100, #ab52917, abcam).

**Data Availability**

The human glioma transcriptomic, genomic, and clinical data were derived from the Ivy Glioblastoma Atlas Project (IvyGap) and TCGA Research Network (<http://cancergenome.nih.gov/>) using TCGAbiolinks. The gene signatures established in this study will be published in MsigDB (<http://www.gsea-msigdb.org/gsea/msigdb>). All other data supporting the findings of this study are available from the corresponding author on reasonable request.
